# Above and Beyond Age: Prediction of Major Postoperative Adverse Events in Head and Neck Surgery

**DOI:** 10.1177/00034894211041222

**Published:** 2021-08-20

**Authors:** Marco A. Mascarella, Nikesh Muthukrishnan, Farhad Maleki, Marie-Jeanne Kergoat, Keith Richardson, Alex Mlynarek, Veronique-Isabelle Forest, Caroline Reinhold, Diego R. Martin, Michael Hier, Nader Sadeghi, Reza Forghani

**Affiliations:** 1 Department of Otolaryngology—Head and Neck Surgery, McGill University, Montreal, QC, Canada; 2 Centre for Clinical Epidemiology, Lady Davis Institute of the Jewish General Hospital, Montreal, QC, Canada; 3 Augmented Intelligence & Precision Health Laboratory (AIPHL) of the Department of Radiology and the Research Institute of McGill University Health Centre, Montreal, QC, Canada; 4 Department of Radiology, McGill University, Montreal, QC, Canada; 5 Department of Geriatric Medicine, Geriatric Institute of Montreal, University of Montreal, Montreal, QC, Canada; 6 Research Institute of the McGill University Health Centre, McGill University, Montreal, QC, Canada; 7 Segal Cancer Centre, Lady Davis Research Institute, Jewish General Hospital, Montreal, QC, Canada

**Keywords:** head and neck cancer, adverse events, surgery, machine learning, frailty

## Abstract

**Objective:**

Major postoperative adverse events (MPAEs) following head and neck surgery are not infrequent and lead to significant morbidity. The objective of this study was to ascertain which factors are most predictive of MPAEs in patients undergoing head and neck surgery.

**Methods:**

A cohort study was carried out based on data from patients registered in the National Surgical Quality Improvement Program (NSQIP) from 2006 to 2018. All patients undergoing non-ambulatory head and neck surgery based on Current Procedural Terminology codes were included. Perioperative factors were evaluated to predict MPAEs within 30-days of surgery. Age was classified as both a continuous and categorical variable. Retained factors were classified by attributable fraction and C-statistic. Multivariate regression and supervised machine learning models were used to quantify the contribution of age as a predictor of MPAEs.

**Results:**

A total of 43 701 operations were analyzed with 5106 (11.7%) MPAEs. The results of supervised machine learning indicated that prolonged surgeries, anemia, free tissue transfer, weight loss, wound classification, hypoalbuminemia, wound infection, tracheotomy (concurrent with index head and neck surgery), American Society of Anesthesia (ASA) class, and sex as most predictive of MPAEs. On multivariate regression, ASA class (21.3%), hypertension on medication (15.8%), prolonged operative time (15.3%), sex (13.1%), preoperative anemia (12.8%), and free tissue transfer (9%) had the largest attributable fractions associated with MPAEs. Age was independently associated with MPAEs with an attributable fraction ranging from 0.6% to 4.3% with poor predictive ability (C-statistic 0.60).

**Conclusion:**

Surgical, comorbid, and frailty-related factors were most predictive of short-term MPAEs following head and neck surgery. Age alone contributed a small attributable fraction and poor prediction of MPAEs.

**Level of evidence:**

3

## Introduction

Despite demographic changes in the incidence of head and neck cancer (HNC), most patients diagnosed with malignancy remain seniors.^1^ Unlike other tumor sites, HNCs often involve critical structures of speech, swallowing, and breathing. The modernization of oncologic treatments has led to improved survival with reduced short and long-term treatment-related toxicities.^2-5^ Despite that, 10% to 45% of patients who undergo radiotherapy or surgery, experience significant adverse events for a cure to be achieved.^6-9^ As more patients become cured of their HNC through surgery, radiotherapy, chemotherapy, or any combination thereof, a significant portion are affected by treatment-related morbidity.^2^ Personalized cancer care, including the appropriate selection of patients for curative-intent treatment, is a growing paradigm in the management of this population.^10-15^

Increasing evidence suggests that frailty, defined as a status of decreased physiologic reserve, is a significant biomarker for survival and postoperative adverse events in oncology patients.^15-17^ Various risk models including several domains of frailty already exist to predict postoperative outcome; however, most are not pragmatic and lack accuracy.^17-19^ The most well-described model in oncology is the Comprehensive Geriatric Assessment (CGA), a multidimensional evaluation of frailty.^20^ Although the CGA provides a rich and thorough evaluation of geriatric oncology patients, it can be resource-intensive and of limited practical use when few therapeutic options exist.^17-19^ Other risk indices, such as the modified frailty index 5 (mFI 5), derived from the American College of Surgeons National Surgical Quality Improvement Program (ACS NSQIP), have quantified frailty based on several physical and comorbid domains.^20-22^ mFI 5 has been shown to be closely associated with postoperative adverse events and prolonged stay in hospital; albeit with limited accuracy.^19-22^ Newer models, incorporating multiple risk factors such as the ACS Universal Risk Calculator, Head and Neck Surgery Risk Index (HNSRI), and Risk Assessment Index (RAI) have allowed for a more personalized and perioperative risk score. These models consider over 15 variables including age.^15,20-22^ Although models with multiple predictor variables can refine cumulative risk, they are often not clinically pragmatic as they add time to collect and are often more challenging to implement in the clinical setting. The importance of perioperative risk prediction cannot be underestimated in a public health system. Firstly, it can help the head and neck surgeon better counsel patients and their families. Additionally, it can aid with postoperative resource allocation, including intensive care unit stay, medical speciality consultation, and discharge planning. The objective of this study was to ascertain which perioperative factors beyond age predict 30-day major postoperative adverse events for patients undergoing head and neck cancer surgery using supervised machine learning. A secondary objective was to evaluate the attributable fraction of age in the prediction of MPAEs in this population.

## Materials and Methods

### Study Design and Population

A retrospective cohort analysis of patients participating in the ACS NSQIP was performed. The ACS NSQIP is a robust, prospectively collected database that records 30-day outcomes of patients undergoing surgery. Patients undergoing non-ambulatory head and neck surgery registered in the NSQIP from 2006 to 2018 were included based on Current Procedural Terminology codes for head and neck surgery (Supplemental Table 1). Pediatric patients, those with perioperative sepsis or ventilator dependence, as well as those undergoing emergency procedures were excluded from the analysis. Ethics approval from the McGill University Health Centre institutional review board was granted (MP-37-2018-3568) for this study.

### Perioperative Clinical Variables

Multiple perioperative clinical variables, including age, sex, smoking status, as well as comorbid cardiac, respiratory, hepatic, renal, and nutritional diseases were recorded. In addition, surgical factors including surgery type, American Society of Anesthesia (ASA) class, operative time, and wound classification were noted. All continuous variables were analyzed as both continuous and subsequently dichotomous variables based on clinically relevant cut-off values.

### Outcome Measure

The primary outcome was a composite measure of major postoperative adverse events (MPAEs) including death within 30 days of index surgery. MPAEs, included death, pulmonary embolism, acute kidney injury, cerebrovascular accident, coma, myocardial infarction, cardiac arrest, sepsis, septic shock, failure to wean off ventilator, re-intubation, multiple blood transfusions, and return to the operating room.^5,7,9,11,12^ These MPAEs have a significant impact on the quality of life of cancer patients and were coded as a binary variable.^5,7,9,10^

### Statistical Analysis

Descriptive statistics were used to evaluate the association between perioperative clinical variables and MPAEs. Multiple imputations of missing data using linear regression were used. Complete case analysis was compared to the data from multiple imputations to dertermine the sensitivity of results. Multiple logistic regression using a stepwise approach to optimize the Bayesian Information Criterion (BIC) was used to construct the regression model. In order to construct machine learning classifiers for the prediction of MPAEs, patients were divided into three groups: a training and a validation cohort using data from 2006 to 2016 and an independent testing cohort using data from 2017 to 2018.^23-29^ The independent testing cohort was used to obtain an unbiased estimate of model generalization error and calculate an unbiased estimate of model performance.^23-29^

Random forests, support vector machines, and neural networks were used as 3 supervised machine learning algorithms for constructing prediction models. Prior to model development, the data was preprocessed by removing uncommon features between the 3 cohorts (training, validation, and testing). Features with more than 50% missing data were also removed. Furthermore, the target variable (major postoperative adverse event) was highly imbalanced across all cohorts (only 11.7% of the patients had undergone a MPAE). Therefore sampling techniques including SMOTE (synthetic minority over-sampling technique) and under-sampling were utilized. Feature selection was performed using recursive feature elimination to identify a discriminative subset of features. Finally, each machine learning algorithm was trained on the subset of selected features and tuned based on their respective hyper-parameters using a 10-fold cross-validation. During the model selection process, the final parameters were selected based on best prediction performance and a compromise between underfitting and overfitting. The logistic and machine learning models were then compared to the HNSRI, ASA and mFI 5 using receiver operating characteristic (ROC) curves. Attributable fractions and delta C-statistics were calculated to assess the contribution of age, both as a continuous and categorical variable, in the prediction of MPAEs.

## Results

A total of 43 701 head and neck surgeries were analyzed with 5106 (11.7%) MPAEs occurring within 30 days of surgery. Table 1 describes the patient characteristic of those in the 2006 to 2016 training and validation cohort. The mean age of this cohort was 56.9 years old with 72.9% of patients being female. There were 3033 free tissue transfer surgeries performed and 14 883 cervical endocrine operations performed. Supplemental Table 2 lists the postoperative adverse events occurring in both cohorts. There were 209 deaths (0.7%) within 30 days of surgery in the training and validation cohort. The most common MPAEs were return to the operating room (5.7%) and bleeding that required multiple transfusion (5.1%).

**Table 1.** Patient Characteristics in the Training and Validation Cohorts.

Factor	Major postoperative adverse event (n = 3846)	No major postoperative adverse event (n = 27 553)
Age, mean (SD)	61.6 (13.8)	56.3 (15.5)
Male, frequency (%)	1399 (62.2)	7091 (43.8)
Hypertension (on medication)	2090 (54.3)	11 613 (42.1)
Dyspnea	532 (13.8)	1794 (6.5)
History of congestive heart failure	64 (1.7)	123 (0.4)
History of COPD	394 (10.2)	1088 (3.9)
Diabetes mellitus	339 (8.8)	1828 (6.6)
Dialysis	54 (1.4)	280 (1.0)
Chronic steroid use	209 (5.4)	794 (2.9)
Disseminated cancer	369 (9.6)	1088 (3.9)
Anticoagulation	198 (5.1)	469 (1.7)
Wound infection	293 (7.6)	440 (1.6)
Current smoker	1158 (30.1)	5214 (18.9)
Preoperative WBC, mean (SD)	8.15 (4.5)	7.27 (2.59)
Preoperative hematocrit, mean (SD)	37.6 (5.9)	40.4 (4.4)
Preoperative serum albumin (g/dL), mean (SD)	3.76 (0.66)	4.08 (0.48)
Weight loss (>10% loss in last 6 mo)	409 (10.6)	501 (1.8)
Functional loss	256 (6.7)	411 (1.5)
American society of anesthesia score		
Class 1	45 (1.2)	1694 (6.1)
Class 2	731 (19)	13 064 (47.4)
Class 3	2554 (66.4)	11 757 (42.7)
Class 4	508 (13.2)	968 (3.5)
Class 5	5 (0.1)	1 (0)
Unknown	3 (0.1)	69 (0.3)
Type of operation		
Neck dissection	609 (15.8)	4160 (15.1)
Salivary	249 (6.5)	3439 (12.4)
Thyroid/Parathyroid	666 (17.3)	14 214 (51.6)
Oral cavity	804 (20.9)	2265 (8.2)
Oropharynx	106 (2.8)	454 (1.6)
Larynx/Hypopharynx	506 (13.2)	1047 (3.8)
Skull base	158 (4.1)	520 (1.9)
Reconstruction	718 18.7)	1301 (4.7)
Other	30 (0.7)	153 (0.6)
Surgical time, minutes (SD)	420.3 (238)	201 (153)
Free tissue transfer	1405 (36.5)	1628 (5.9)
Tracheostomy	752 (19.6)	914 (3.3)
Wound classification		
Clean	1274 (33.1)	19 714 (71.5)
Clean-contaminated	2338 (60.8)	7427 (27)
Contaminated	145 (3.8)	280 (1.0)
Dirty	89 (2.3)	132 (0.5)

Abbreviations: COPD, chronic obstructive pulmonary disease; SD, standard deviation; WBC, white blood cell count.

**Figure 1.**
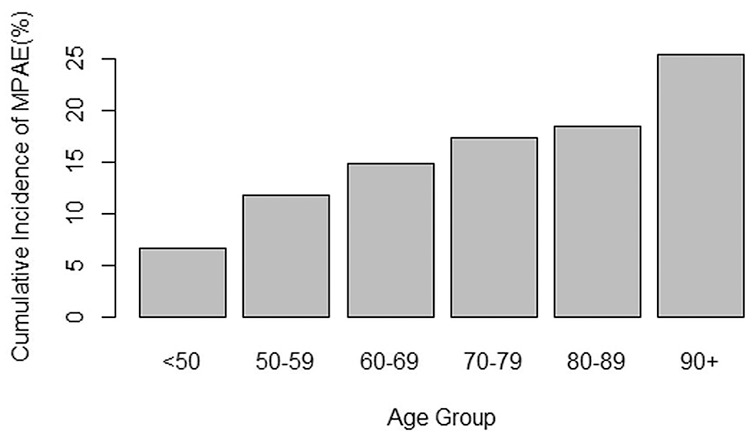
Cumulative incidence of short-term major postoperative adverse events by age group. *Note.* Distribution of cumulative incidence of 30-day major postoperative adverse events by age group.

Using multiple logistic regression, the retained predictor variables are listed in Table 2. The largest attributable fractions associated with MPAEs were for ASA class 3+, hypertension on medication, prolonged operative time, sex, preoperative anemia, and free tissue transfer used. Age both as a continuous and a categorical variable, showed statistical association with MPAEs in both univariate and multivariate logistic regression (Figure 1, Table 3). Moreover, the attributable fraction of age on predicting MPAE was small compared to other variables (Table 3).

**Table 2.** Retained Perioperative Factors Using Supervised Machine Learning and Multiple Logistic Regression to Predict Major Postoperative Adverse Events.

Model	Predictor variables	Association to MPAE	Prevalence (%)	Population attributable fraction
(Performance score*)				
Supervised machine learning	Operative time (>500 min)	29.6	10.0	—
	Anemia (hematocrit <35)	23	10.6	—
	Free tissue transfer	22	10.0	—
	Recent weight loss	13.6	2.9	—
	Wound classification	13.6	2.1	—
	Hypoalbuminemia (<3.5 g/dL)	9.7	4.9	—
	Preoperative wound	9.3	2.4	—
	Tracheotomy (concurrent)	8.5	5.5	—
	ASA Class 3+	7.8	50.2	—
	Sex (male)	5.9	45.5	—
(Odds ratio)				
Multiple logistic regression	ASA Class 3+	1.54 (1.19-2.00)	50.2	21.3
	Hypertension on medication	1.43 (1.21-1.69)	43.5	15.8
	Operative time (>500 min)	2.81 (2.26-3.50)	10.0	15.3
	Sex (male)	1.33 (1.12-1.58)	45.5	13.1
	Anemia	2.38 (1.95-2.90)	10.6	12.8
	Free tissue transfer	1.99 (1.57-2.52)	10.0	9.0
	Laryngectomy/pharyngectomy	1.92 (1.32-2.81)	5.7	5.0
	Tracheotomy (concurrent)	1.84 (1.43-2.38)	5.5	4.4
	Wound classification	2.49 (1.51-4.09)	2.1	3.0
	Dyspnea	1.33 (1.04-1.70)	8.3	2.7
	Hypoalbuminemia	1.51 (1.21-1.89)	4.9	2.4
	Recent weight loss	1.82 (1.35-2.44)	2.9	2.3
	Anticoagulation	1.97 (1.36-2.84)	2.1	2.0
	Chronic steroid use	1.43 (1.03-1.98)	3.2	1.4
	Functional status	1.60 (1.09-2.34)	2.0	1.2

*Note.* Retained perioperative factors most predictive of major postoperative adverse events (MPAE) in this population.

Abbreviation: ASA, American Society of Anesthesia.

**Table 3.** Age as a Predictive Factor Using Supervised Machine Learning and Logistic Regression.

Analysis method	Association to MPAE	Prevalence (%)	Population attributable fraction (%)
(Performance score*)			
Supervised machine learning		—	—
Age <50	4.22		
Age 80-89	1.71		
Age 70-79	1.64		
Age 50-59	0.97		
Age 60-69	0.71		
Age 90+	0.52		
(Odds ratio)			
Multiple logistic regression			
Age (continuous)	1.01 (1.00-1.02)	—	—
Age <50	REF	29.9	—
Age 50-59	0.96 (0.74-1.25)	26.6	1.0
Age 60-69	1.13 (0.86-1.47)	23.6	2.8
Age 70-79	1.34 (1.00-1.79)	13.2	4.3
Age 80-89	1.22 (0.85-1.74)	6.3	1.4
Age 90+	2.44 (0.90-6.61)	0.4	0.6

*Note.* Association of age with 30-day major postoperative adverse event (MPAE).

Abbreviation: REF = Reference.

Using supervised machine learning, the most significant perioperative variables associated with MPAEs having the highest weights were prolonged operative time (over 500 minutes), preoperative anemia, free tissue transfer, preoperative weight loss, clean-contaminated wound classification, preoperative hypoalbuminemia, preoperative wound infection, tracheotomy (concurrent), ASA class 3 or greater, and sex. Age both as a continuous and categorical variable did not have a significant contribution in predicting MPAEs. The inclusion of 10 features in the random forests supervised machine learning model developed the largest delta C-statistic (Table 3).

The area under the curve (AUC) of the supervised machine learning (SML) model to predict MPAEs using the independent test cohort was 0.846 (95% CI of 0.837-0.85) as compared to 0.855 (0.847-0.864) for the multiple logistic regression model (Table 4). The addition of age to the SML, logistic or ASA class models did not show any significant increase in AUC as compared to the mFI 5 (Table 4).

**Table 4.** Comparison of Models to Predict Major Postoperative Adverse Events in the Independent Testing Cohort.

Model	AUC	ΔC age (categorical)*	ΔC age (continuous)**
Supervised machine learning	0.846 (0.837-0.855)	0.001 (*P* = .92)	0.004 (*P* = .75)
Multiple logistic regression	0.855 (0.847-0.864)	0 (*P* = 1.00)	0.003 (*P* = .91)
American society of anesthesia score	0.693 (0.686-0.700)	0.007 (*P* = .09)	0.016 (*P* = .06)
Modified frailty index 5	0.592 (0.585-0.600)	0.035 (*P* < .001)	0.037 (*P* < .001)

Abbreviation: AUC, area under the curve.

Age* as a categorical variable.

Age** as a continuous variable.

## Discussion

Identifying patients at high risk of adverse outcomes following head and neck cancer surgery can help guide management without necessarily compromising oncologic success. Beyond counseling patients and their families, simple and accurate prediction of postoperative outcome may help head and neck surgeons allocate postoperative resources including intensive care unit monitoring and discharge planning to an intermediate care facility. Such a risk score may identify patients at heightened risk for surgery who may benefit from pre-habilitation. Consequently, chronological age, any patient can have a high-risk profile and is more likely to have a MPAE.

The supervised machine learning and logistic regression models had similar accuracy and outperformed the modified frailty index 5 and ASA class. Although these risk models carry similar features to the HNSRI, the methods to identify risk factors as well as weights assigned to predictor variables are intrinsically different. In the HNSRI, the highest risk was attributed to prolonged operative time (over 8 hours), dirty surgical wound sites, preoperative anemia, and age 90 or above.^15^ Interestingly, age by itself did not have sufficient predictive ability for MPAEs compared to other perioperative variables to be included in the final model, despite modeling age as both a categorical and continuous variable. Moreover, the population attributable fraction of age was significantly lower than other predictor variables, including ASA score, hypertension on medication, prolonged operative time, sex, preoperative anemia, and free tissue transfer use. This further emphasizes the importance of multi-level clinical variables beyond age alone. Several studies have shown that chronological age in itself is not a risk factor for poor survival or adverse event following oncologic treatment.^8,9^ Seemingly, physiologic age defined by comorbid status, frailty, and sarcopenia more accurately represents risk of poor outcome following cancer treatments.^10,21,22^ These clinical variables extend beyond surgical factors and include sarcopenia biomarkers. Although no cause-effect model was developed, it may be further considered if modifications of such perioperative high-risk factors can mitigate postoperative risk of adverse events.

This study is limited by selection and measurement bias. In particular, the NSQIP dataset includes surgical patients only. In order to develop a larger predictive ability, a heterogeneous group of patients undergoing head and neck surgery were included; the majority of patients in our cohort underwent cervical endocrine operations. Additionally, indications for surgery as well as a description of patients who did not undergo surgery is not available. No data is available on TNM staging, previous cancer treatments or use of chemoradiation of the head and neck. More recent datasets from the NSQIP are available with tumor-related information for thyroid surgery. This data may be further used to quantify major adverse events following thyroid surgery specifically. Additionally, outcomes in this dataset are limited to 30 days from index surgery with no head and neck cancer specific functional outcomes nor survival related to treatment. Although, the data was internally tested using an independent test set; an external set of prospectively collected data would be required to confirm generalizability.

## Conclusion

Surgical, comorbid, and frailty-related risk factors had the largest attributable fraction and were most predictive of short-term major postoperative adverse events following head and neck surgery in the NSQIP database. Age was identified as an independent prognostic factor; however, with a small attributable fraction and risk prediction. The decision to perform head and neck surgery on seniors should not be affected by the patient’s age alone.

## Supplemental Material

sj-pdf-1-aor-10.1177_00034894211041222 – Supplemental material for Above and Beyond Age: Prediction of Major Postoperative Adverse Events in Head and Neck Surgery Click here for additional data file. Supplemental material, sj-pdf-1-aor-10.1177_00034894211041222 for Above and Beyond Age: Prediction of Major Postoperative Adverse Events in Head and Neck Surgery by Marco A. Mascarella, Nikesh Muthukrishnan, Farhad Maleki, Marie-Jeanne Kergoat, Keith Richardson, Alex Mlynarek, Veronique-Isabelle Forest, Caroline Reinhold, Diego R. Martin, Michael Hier, Nader Sadeghi and Reza Forghani in Annals of Otology, Rhinology & Laryngology
