# Supplementary material for: Above and Beyond Age: Prediction of Major Postoperative Adverse Events in Head and Neck Surgery
Source: Ann Otol Rhinol Laryngol. 2021 Aug 20;131(7):697–703. doi: 10.1177/00034894211041222 (PMC9203666; doi:10.1177/00034894211041222)
Supplement: sj-pdf-1-aor-10.1177_00034894211041222 – Supplemental material for Above and Beyond Age: Prediction of Major Postoperative Adverse Events in Head and Neck Surgery [file sj-pdf-1-aor-10.1177_00034894211041222.pdf]

Supplemental Tables

Supplemental Table 1 – Current Procedural Terminology codes for head and neck surgery

**Organ System**	**Procedure**	**CPT** (malignant)
Larynx/upper trachea	Laryngectomy	31365, 31367, 31368, 31370, 31375, 31380, 31382, 31390, 31395, 31420, 31785
	Suspension microlaryngoscopy	31400
Neck	Dissection	38542, 38700 38720, 38724
Oral Cavity		40816, 40814, 40812, 40810, 40818
	Tongue	41112, 41113, 41114, 41110, 41116, 41135, 41140, 41145, 41150, 41153, 41130, 41120, 41155
	Mouth (other)	41827, 41826, 41825,
Parotid	Parotidectomy	42415, 42410, 42420, 42425, 42426
	Submandibular Gland	42440
Cutaneous		
	External auditory Canal	69150, 69155, 69145
	Face/Head/Neck	11440,11446, 11640,11641, 11642, 11643, 11644, 11645, 11646, 11420,11426, 11620,11621, 11622, 11623, 11624, 11626, 21011, 21012, 21013, 21014,21015, 2101621552, 21554, 21555, 21556, 21557, 21558, 30150, 30160, 40500, 40530
	Lip	40527, 40525, 40510, 40520, 40530, 40500
Endocrine	Thyroid	60210, 60212, 60220, 60225, 60240, 60252, 60254, 60260, 60270, 60271
	Parathyroid	60500, 60502, 60505, 60512
	Carotid Body Tumor	60605, 60600
Skull Base	Craniectomy	61518, 61548, 61580, 61581, 61582, 61583, 61584, 61585, 61590, 61591, 61592, 61595, 61600, 61601, 61605, 61606, 61607, 61608, 67412, 67420, 61618
	Maxilla/ethmoid	31230, 31225, 31205, 31200, 31201
Hypopharynx		43100, 43287, 43288, 43286
Reconstruction		14020, 14021, 14040, 14041, 15756, 15757, 15758, 20956, 20955, 20962,20970, 20969, 43496
	Adjacent tissue	14060, 14061, 14301, 14302
	Loco-regional + graft	15630, 15620, 15610, 15600, 15731, 15576, 15574, 15572, 15570, 15730, 15733, 15734, 15736, 15650, 15740, 15750, 15760, 15770

	Free	15758, 15756, 15757
Pharynx		
	Tonsil/Base of tongue	42842, 42844, 42845
	Hypopharynx	42890, 42892, 42894
Tracheotomy		31600, 31603, 31610

CPT: Current Procedural Terminology

Supplemental Table 2 – Postoperative adverse events in both the derivation and validation cohorts

Postoperative Adverse Event	***Derivation Cohort*** *(n=31399)*	***Validation Cohort*** *(n=12302)*
**Death***, (%)	209 (0.7)	57 (0.4)
**Surgical Adverse Events**, (%)		
Superficial wound infection	647 (2.1)	269 (2.2)
Deep space infection*	333 (1.1)	99 (0.8)
Wound Dehiscence	393 (1.3)	148 (1.2)
**Cardiovascular Adverse Events**, (%)		
Acute Myocardial Infarction*	114 (0.4)	50 (0.2)
Cardiac Arrest*	99 (0.3)	29 (0.2)
**Respiratory Adverse Events**, (%)		
Pneumonia	568 (1.8)	186 (1.5)
Failure to wean off ventilator*	446 (1.4)	105 (0.9)
Reintubation*	371 (1.2)	136 (1.1)
**Renal Adverse Events**, (%)		
Progressive Renal Insufficiency	21 (0.07)	10 (0.08)
Acute Renal Failure*	35 (0.1)	6 (0.05)
**Infectious Adverse Events**, (%)		
Urinary Tract Infection	213 (0.7)	57 (0.4)
Sepsis*	328 (1)	73 (0.6)
Septic Shock*	73 (0.2)	24 (0.2)
**Neurological Adverse Events**, (%)		
Stroke*	72 (0.2)	26 (0.2)
**Hematologic Adverse Events**, (%)		
Bleeding requiring transfusion*	1596 (5.1)	516 (4.2)
Pulmonary Embolism*	101 (0.3)	34 (0.3)
Deep Vein Thrombosis	140 (0.4)	55 (0.5)
**Return to OR***, (%)	1799 (5.7)	661 (5.4)

OR: Operating Room. * indicates a major postoperative adverse event
